# The performance of NLST screening criteria in Asian lung cancer patients

**DOI:** 10.1186/s12885-015-1922-5

**Published:** 2015-11-18

**Authors:** Vivek Kumar, Kevin Becker, Huo Xiang Zheng, Yiwu Huang, Yiqing Xu

**Affiliations:** 1 Department of Internal Medicine Maimonides Medical Center, 4802 10th Avenue, Brooklyn New York, 11219 USA; 2 Department of Hematology and Oncology Maimonides Cancer Center, 6300 Brooklyn, New York, 11220 USA

**Keywords:** NLST criteria, Performance, Lung cancer, Asian, Screening, Validity, Extrapolation, Never, smokers

## Abstract

**Background:**

Screening high-risk individuals with low dose CT decreased lung cancer mortality in the National Lung Screening Trial (NLST), but the validity of directly extrapolating these results to an Asian population is unclear. Using statistical models on Surveillance, Epidemiology and End Result (SEER) data, 27 % of lung cancer patients in the United States were estimated to meet the screening criteria. This study aims to evaluate the performance of the NLST criteria in Asian lung cancer patients and to examine the characteristics of those who did not meet the criteria.

**Methods:**

We conducted a retrospective study of Asian lung cancer patients treated at Maimonides Cancer Center between 1/2008 and 6/2013. Data on demographics, smoking history, cancer stage, histology, and EGFR/ALK mutation status were collected and analyzed.

**Results:**

Of 116 eligible patients, 75 patients (65 %) were smokers which included 26 light smokers (22 %). Thirty-two patients (27.8 %) met the NLST criteria. Extending the age limit to 79 would cover 8 % more patients while removing the lower age limit would only cover 2 % more. None of the female patients met the criteria as they were all never or light smokers. Two-thirds of male patients younger than age 55 were never or light smokers. The EGFR mutation rate was 67 % in female and 28 % in male patients.

**Conclusion:**

The percentage of Asian patients meeting the NLST criteria is similar to that estimated for the United States population, suggesting that extension of the criteria to an Asian population is valid. One-third of the patients were non-smokers and an additional one-fourth were light smokers, comprised mostly of female and young male patients. Further strategies for screening these individuals based on non-tobacco factors are urgently needed.

## Background

Lung cancer is the most common cancer in the world. According to the World Health Organization (WHO), approximately 1.8 million new cases were diagnosed worldwide in 2012, 58 % of which occurred in Asia and Africa [1]. While the incidence of lung cancer is decreasing in the United States, it continues to grow in Asia [2]. The 5-year survival rate is only 49 % in stage I, and a dismal 2 % in stage IV disease [3]. The recently published National Lung Screening Trial (NLST) showed a 20 % reduction in lung cancer mortality after three rounds of annual screening by low dose CT in comparison to conventional chest radiography [4]. The entry criteria included 1) age 55–74 years, 2) a history of smoking at least 30 pack-years and 3) currently smoking or quit smoking within 15 years. These high-risk criteria emphasize age and cumulative smoke exposure. Based on these numbers and the projection of subjects aging during the screening period in the NLST, the United States Preventive Services Task Force (USPSTF) recommends screening individuals age 55–80 with similar smoking exposure [5].

It has been demonstrated that smoking is responsible for up to 90 % of cases of lung cancer in developed countries, with the risk increasing with quantity and duration of smoking [6]. However, the epidemiology of lung cancer may be different in Asian populations [7]. While the prevalence of smoking [8], air pollution and environmental hazards [9] are considered to be significantly higher in developing countries, up to 30–40 % of Asian lung cancer patients are never smokers in contrast to only 10 % of patients in the United States [10].

Lung cancer in Asians is also genetically diverse with up to 35 % of patients harboring epidermal growth factor receptor (EGFR) mutations in contrast to only 10 % of Caucasian patients [11, 12]. The EGFR belongs to the receptor tyrosine kinase (RTK) family. The binding of ligands, such as epidermal growth factor, induces a conformational change that leads to receptor homo- or heterodimer formation, which results in activation of EGFR tyrosine kinase activity. Activated EGFR then phosphorylates its substrates, resulting in activation of multiple downstream pro-survival pathways involved in cell proliferation. Approximately 90 % of these mutations are exon 19 deletions or exon 21 L858R point mutations [13]. Another important mutation, the anaplastic lymphoma kinase (ALK) translocation, is responsible for approximately 3–5 % of non-small-cell lung cancer (NSCLC) and is found predominately in adenocarcinomas [14]. ALK lung cancer patients are likely to be younger and never or light smokers. ALK-rearrangements in NSCLC are, for the most part, not found in EGFR- or KRAS-mutated tumors [15]. These mutations provide therapeutic targets for several tyrosine kinase inhibitors.

In the NLST trial, only 2 % of the study population were Asian, presumably diluting the effect of characteristics found only in Asian patients [4]. Using statistical models on data derived from Surveillance, Epidemiology and End Result (SEER), Pinsky et al. calculated that 27 % of lung cancer patients in the United States would have met the criteria for screening [16]. We hypothesized that fewer existing Asian lung cancer patients would have met these screening criteria. In this study, we aimed to evaluate the performance of the NLST criteria on existing Asian lung cancer patients; we also focused on the assessment of the characteristics of those not meeting the criteria.

## Methods

We conducted a retrospective chart review of Asian lung cancer patients diagnosed or treated at Maimonides Cancer Center in Brooklyn, New York between 1/2008 and 6/2013. The study was reviewed and approved by the Institutional Review Board (IRB) of Maimonides Medical Center. The study participants were all adults. The consents were waived by IRB as it was a retrospective study. Cases were identified from the electronic medical record and Asian ethnicity was determined after a multistep process. The Asian patients in the current study were born in Asia, mostly in China, Pakistan and Bangladesh, and migrated to United States later in life. Ethnicity was determined initially by last name. It was further confirmed by reviewing medical records documentation. This documentation was done by two Chinese speaking physicians, YH and YX, after interviewing the patients during the original encounter. This multi-step process ensured that the ethnicity was recorded as accurately as possible. We collected data on demographics, cancer stage at diagnosis, histology, history of smoking including number of pack years and time since quitting, and EGFR/ALK mutation status. We calculated the percentage of patients who would have been eligible for screening based on the NLST criteria: 1) Age 55–74 years old, 2) History of smoking at least 30 pack-years and 3) Current smokers or quit within 15 years. We then analyzed the characteristics that would have excluded patients from the screening recommendations. For this study “Never smokers” were defined as people who smoked <100 cigarettes in their life time. The “light smokers” were defined as people who smoked ≤ 10 pack-years and those who smoked > 10 pack-years were labeled as “heavy smokers” [24]. These definitions are similar to those used by other epidemiological studies [30–32].

## Statistical analysis

The sample size calculation was performed using SEER data as a comparison. We hypothesized that among Asian lung cancer patients, only 10 % would have met the screening criteria, as compared to 27 % based on SEER data. Using a 95 % confidence limit of +/- 5 %, 139 patients were required with a precision/absolute error of 5 % and a type I error of 5 %. After exclusion of non eligible patients, our actual sample size was 116. It was low to find any small difference between the two groups; however, it could have detected a large difference. In an exploratory analysis, Chi-square testing was applied to detect a significant difference between the result in our study and that in the literature.

## Results

One hundred fifty-one patients were identified; 35 patients were excluded due to incomplete information. Of the 116 patients included in the analysis (Table 1), the median age was 66 years with a range of 33–92 years. Seventy-seven patients (67 %) were male and 39 (33 %) were female. Most patients were diagnosed with advanced disease: 20 patients (17 %) had stage III disease and 65 patients (56 %) had stage IV disease. A minority of patients (29 patients, 25 %) were diagnosed with stage I and II diseases. The most common histology was adenocarcinoma in 74 patients (64 %).

**Table 1:** Characteristics of the study population

Total patients	*N* = 116 (100 %)	Smoking status	
Male	77 (66.38 %)	Never smokers	41
Female	39 (33.62 %)	Male	5
Ethnicity		Female	36
Chinese	106 (91.38 %)		
Indian subcontinent^a^	10 (8.62 %)		
Age		Ever smokers	75
Age range	33–92 years	Heavy smokers	
Median age	66 years	Male	54
Age < 55 years	18	Female	0
Age 55–74 years	69	Light smokers	
Age > 74 years	29	Male	18
		Female	3
Histology		Stages	
Adenocarcinoma	74 (63.8 %)	Stages I and II	29
Squamous cell	24 (20.7 %)	Stages III and IV	85
Small cell	8 (6.9 %)	Unknown	2
Other rare variants	10 (8.6 %)		
EGFR status		ALK mutation status	
Positive	24/53	Positive	0/33
Male	8/29	Negative	33/33
Female	16/24		

^a^Immigrants from Pakistan and Bangladesh

**Fig. 1:**
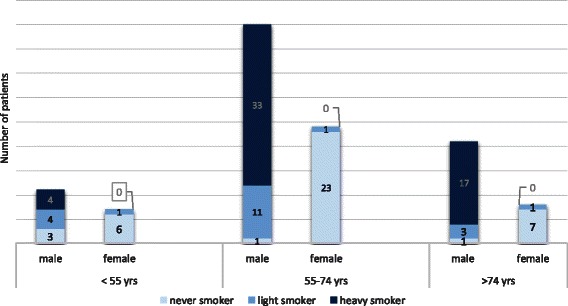
Distribution of heavy smokers/light smokers/never smokers in different age group

All patients were Asian born who immigrated to the U.S. as adults. One hundred six (91 %) patients were of Chinese origin and 10 patients (9 %) migrated from the Indian Subcontinent. Seventy-five patients (65 %) were smokers (72 male and 3 female), of which, 26 patients (22 %) were light smokers. The other 41 patients (35 %) were never smokers. The distribution of heavy, light and never smokers by gender and age group is depicted in Fig. 1 and Table 2. Among never smokers, 36 were female and 5 were male (Table 2). Among 39 female patients, 36 were never smokers and 3 were light smokers (Table 2, and Fig. 1).

**Table 2:** Comparison of patients meeting NLST criteria between US population and Asians

Age groups	55–74 years	55–79 years	55–74 years
Smoking status	30 + pack-years current or quit < 15 years	30 + pack-years current or quit < 15 years	Ever smokers
Native U.S. population^a^	26.7 %	32.9 %	47.9 %
Asians	27.8 %	35.3 %	38.8 %
*P value*	>0.05	> 0.05	0.03

^a^Estimated by Pinsky et al. on data derived from SEER and US census [16]

On applying the NLST criteria, 32 patients (27.8 %) would have met the recommended screening criteria (Table. 2). This represents 48 % of patients in the age range of 55–74 years. (Table 2). Twenty nine patients were older than 75 years (25 %); twelve of those patients were heavy smokers who would have otherwise fit into the NLST criteria. By extending the upper age limit to 79 years, an additional 9 patients (8 %) would have met the criteria (Table 2). However, in the group of patients younger than 55, only 4 out of 18 male patients were heavy smokers and 3 of them would have otherwise met the NLST criteria (2 % more) (Table 2). Interestingly, in this age group, 3 male patients were never smokers and 4 were light smokers. This indicates that lowering the age limit alone may not significantly increase the number of cancers detected.

None of the female patients met the screening criteria based on smoking history: Thirty-six were never smokers and 3 were light smokers. (Table 2, Fig. 1). An exploratory analysis was performed to evaluate the increase in cases meeting screening criteria by relaxation of the age limits as well as the smoking history in this study, and to compare our results to that extracted from the study on SEER data [16] as shown in Table 2. By extending the upper age limit to 79 years, 32.9 % of Caucasian patients would be covered, and similarly 35.3 % of our patients were covered (*p* > 0.05). By including all ever smokers in the 55–74 age group, 47.9 % U.S. lung cancer patients would be covered, while only 38.8 % of Asian patients would be covered, this difference was statistically significant (*p* = 0.03).

**Fig. 2:**
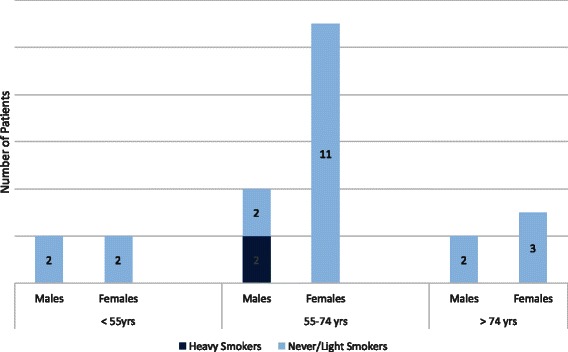
Distribution of EGFR mutations in different age groups

EGFR mutation analysis was available for 53 patients, 24 were positive for exon 19 or exon 21 mutations (Fig. 2). The EGFR-mutated cancers were seen in 16 out of 24 tested female patients (67 %), all light or never smokers. EGFR mutation was detected in 8 out of 29 tested male patients (28 %) and 6 were light or never smokers while 2 were heavy smokers who actually met the NLST screening criteria (Table 1). The distribution of EGFR positive cases in the three age groups according to gender and smoking history is depicted in Fig. 2. The EGFR mutation positive cases distributed evenly in all three age groups in both male and female patients (Fig. 2). Thirty three patients were tested for ALK translocation but none was positive for this mutation.

## Discussion

The NLST criteria were established after study of a general U.S. population; however, direct extrapolation to Asian patients warrants further consideration. The epidemiology of lung cancer in Asian populations is different, including more smokers [17] in the community leading to greater second hand smoking exposure as well as other environmental hazards [18, 19]. In addition, a higher percentage of EGFR-mutated cancers are observed in Asians patients than in the general U.S. population [20], which correlates with the higher percentage of never smokers among lung cancer patients in Asian countries. It is imperative to develop screening criteria that take into account the heterogeneous risk factors in Asian populations.

In our retrospective study of already diagnosed Asian lung cancer patients, 27.8 % would have met the NLST criteria for screening. The result rejected our hypothesis and revealed that there is no large difference in the rate of meeting the screening criteria between Asian lung cancer patients and that of the U.S. population as estimated in the study published by Pinsky et al. [16] In that study, calculations were based upon statistical models using data derived from SEER, the U.S. Census and the National Health Interview Survey, and found that in the United States 27 % of lung cancer patients would have met the criteria for screening [16]. In the present study, our data suggest that the performance of the NLST criteria to detect lung cancers in an Asian population is similar to the general U.S. population. It also implies that the proportion of lung cancer attributable to heavy smoking in patients aged 55–74 years is extremely similar in the Asian and general U.S. populations. Smoking is still the strongest risk factor for lung cancer in Asian populations.

However, if smoking is presumed to be the key risk factor for lung cancer, then broadening the smoking eligibility should detect more lung cancer patients. The study by Pinsky et al. [16] suggested that broadening the current criteria to ever smokers in the 55–74 age range would cover 47.9 % of patients. However this modification may help only marginally to boost the coverage rate in Asian patients as only 37 % of the patients in our study would fulfill these criteria. This difference was statistically significant. In addition, while the coverage could increase by 8 % if the upper age limit was increased, reducing the lower age limit would only marginally increase coverage by 2 %. The smoking behavior in patients younger than age 55 revealed in this study merits further attention. Two thirds of the male patients and all of the female patients in this group were never or light smokers. Carcinogens other than tobacco are likely to be the driving force and require further study. Furthermore, 35 % of our patients were never smokers and all of our female patients were never smokers or light smokers. This data is consistent with previous reports [22]. It is quite different from studies in the U.S. where smoking accounts for up to 90 % of lung cancers and only 19 % of female lung cancer patients report no smoking history [21]. Overall, our study has highlighted an important difference in the percentage of Asian lung cancer patients who are never smokers or light smokers. Relaxing the stringent smoking history in the NLST criteria will still not help this cohort.

The development of screening criteria for never or light smokers is a great challenge. In view of the vast number of patients with lung cancer in Asia, 30–40 % of whom are never smokers, this is a serious problem [23]. Theoretically, the risk factors in this group could be environmental hazards, the development of driver mutations in the EGFR or other genes, or an interaction between the two. There is a high EGFR mutation rate in both male and female patients in this study, consistent with that reported in previous studies of Asian patients [24]. This holds promise as potential screening marker in never/light smokers in the future. There has been persistent interest in detecting molecular signatures in the sputum [25], bronchial alveolar lavage and peripheral blood [26] of lung cancer patients, albeit with little success. Those studies utilized FISH for EGFR mutations [27], micro RNA [28] and specific gene methylation markers [29]. Besides being non-invasive, assays for EGFR mutation analysis should be at least as sensitive but more specific than low dose CT to minimize the risk of false positive results, a major shortcoming of CT screening in the NLST trial. In an encouraging small study testing this model, the sensitivity of EGFR mutation detection in the sputum using various techniques varied from 30–50 %, with very high specificity of up to 100 % [25].

To our knowledge, this is the first study to evaluate the performance of the NLST criteria in Asian patients. The strength of this study is the detailed analysis of an array of factors including smoking history, duration, age, EGFR status, etc. in a unique population who are of Asian descent. The limitations of this study are the small sample size which would not allow detection of a small difference if it existed. In addition, this is a retrospective study, so patients whose smoking history was incompletely documented were excluded. The other drawback is the lack of information on the length of time residing in the U.S., a variable that could affect hazardous exposures. Finding a similar percentage of Asian patients with lung cancer who met the criteria suggests that Asian patients may derive a survival benefit from screening similar to that of the general U.S. population studied in the NLST. It is important to have a randomized clinical trial dedicated to Asian lung cancer patients to confirm such a benefit.

## Conclusion

The percentage of Asian patients with lung cancer who would have met the NLST screening criteria was not largely different from that estimated for the general U.S. population. The NLST criteria which emphasize tobacco exposure are valid for Asian patients. One third of Asian lung cancer patients are never or light smokers and consist mainly of female patients and those below the age of 55. Further studies are needed to identify additional risk factors beyond smoking alone to construct a more comprehensive screening model for the early diagnosis of lung cancer in Asian populations.

## Abbreviations

ALC: Asian lung cancer
EGFR: Epidermal growth factor receptor
NLST: National lung screening trial
SEER: Surveillance, epidemiology and end result
USPSTF: United States Preventive Services Task Force
